# Study on a Hydrogel for Adsorption of Chloride Ions in Cementitious Materials

**DOI:** 10.3390/polym14102081

**Published:** 2022-05-20

**Authors:** Meng Cao, Lili Wu, Guixia Zhang, Ying Yang, Wei Chen, Qiu Li, Pei Tang, Wanyu Chen

**Affiliations:** 1 State Key Laboratory of Silicate Materials for Architectures, Wuhan University of Technology, Wuhan 430070, China; caomeng19970705@163.com (M.C.); chen.wei@whut.edu.cn (W.C.); qiuli@whut.edu.cn (Q.L.); pei-tang@whut.edu.cn (P.T.); 2 School of Materials Science and Engineering, Wuhan University of Technology, Wuhan 430070, China; polymer_wl@whut.edu.cn (L.W.); zhang94670@whut.edu.cn (G.Z.); yingyang2006@163.com (Y.Y.)

**Keywords:** polyacrylamide hydrogel, chloride ion, adsorption, cementitious materials

## Abstract

Chloride ions in the seaside environment can corrode the steel reinforcement in concrete, which greatly endangers the safety of seaside structures. As an excellent adsorption material, hydrogel is widely used in the field of water treatment but is rarely used in cementitious materials. In this study, a polyacrylamide–chitosan hydrogel (PAMC) was prepared with N,N-methylenebisacrylamide as the cross-linking agent and acrylamide as the monomer. The prepared PAMC gel could effectively adsorb chloride ions in simulated seawater and simulated sea sand environments, and the maximum adsorption capacity of chloride ions by PAMC-1 (prepared from 2.5 g acrylamide and 1% content of N,N-methylenebisacrylamide relative to acrylamide) gels in simulated seawater was 55.53 mg/g. The adsorption behavior of the PAMC gels in solution fit the Langmuir isotherm model. The composition and morphology of the PAMC gel were characterized, and the responsiveness of the PAMC gel to the environment was studied. The results showed that the PAMC gels adsorbed better in alkaline environments and thus could be used in alkaline cement-based environments. The mortar sample containing the PAMC-1 gel had higher resistance to chloride ion penetration, and the chloride ion content at 7.5–10mm from the surface of the sample cured for 28 days was reduced by 41.4% compared to the samples without the gel.

## 1. Introduction

A hydrogel is a polymer with a three-dimensional network structure [1,2] and a controllable structure that allows the gel to respond to the environment by adjusting the functional groups on the chain segments. Because of the porous structure and electrostatic interactions [3,4], hydrogen bonding [5], and chemical interactions between functional groups and solutes, hydrogels have been widely employed as adsorbents in many areas such as ion adsorption [6,7], organic pollutant adsorption, and dye adsorption [8]. The anion adsorption function of the gel is commonly used in water treatment, such as the removal of phosphate and arsenate [9,10] from wastewater or fluoride and perchlorate [11] from drinking water. Zhuolin Qing et al. [12] prepared pH-sensitive sodium alginate/zirconium (SA/Zr) hydrogels by directly dropping sodium alginate solution into zirconium chloride solution to crosslink. When the pH value was less than 4.19, the SA/Zr hydrogel adsorbed phosphate by electrostatic attraction and hydrogen bonding, and the gel reached the maximum adsorption capacity (256.79 mg PO_4_^3−^/g) at pH = 3.

With the vigorous development of marine engineering such as cross-sea bridges and submarine tunnels, the concern of durability and safety of concrete materials in seaside environments had been raised [13]. Chlorine ion attack, microbial corrosion, carbonation, sulfate attack, etc., are the main factors affecting the durability of seaside buildings. Among them, chloride ions entering the concrete interior through infiltration and capillary adsorption [14,15] in the seaside environment could locally lower the pH value of the reinforcing steel surface to below 4. The low pH would destroy the passivation film to cause rusting of the reinforcing steel [16,17,18,19], which damaged the structural stability and durability of marine buildings. Therefore, effective adsorption and solidification of chloride ions in reinforced concrete was particularly important to optimize the structural safety and extend the service life of marine buildings.

Concrete structures are porous and prone to cracking, which accelerates the transport of chloride ions. Water molecules, oxygen, carbon dioxide, and chloride ions in the air can all be effectively prevented from entering the concrete by coating the building surface. Guo Li [20] synthesized a novel nano polymer-modified cementitious (PMC) coating by adding nano-SiO_2_ or nano-TiO_2_ suspension to acrylic emulsion. The nano-SiO_2_ or nano-TiO_2_ suspension can reduce the porosity of the coating, and the service life of the coated concrete against chloride attack can be extended by 7.1 times or 2.8 times with the addition of 0.5% nano-SiO_2_ or nano-TiO_2_ to the PMC coating. In addition, there are methods such as crack reduction [21], cathodic protection [22], steel bar anticorrosion, and addition of corrosion inhibitors [23]. These methods can retard or impede the chloride ion transport process.

In order to further hinder and cure the chloride ions that have penetrated into the interior of concrete, a polyacrylamide–chitosan semi-interpenetrating network (PAMC) hydrogel was synthesized in this paper. The PAMC can trap chloride ions in simulated seawater and sea sand environments using its swelling performance and the hydrogen bonds formed by the functional groups on the polymer chains with chloride ions. The gels were characterized by infrared spectroscopy (IR), scanning electron microscopy (SEM), and energy disperse spectroscopy (EDS) to study their adsorption mechanism for chloride ions in simulated seaside environments and their responsiveness to pH value. The ability of the gel to solidify chloride ions when they enter the mortar through external infiltration was investigated and quantified. The chloride ion content of mortar samples curing for 14 days at 7.5–10 mm from the surface was reduced by 55.8% and that of mortar samples maintained for 28 days at 7.5–10 mm from the surface was reduced by 41.4% when PAMC-1 was dosed at 1.06 wt%.

## 2. Experimental

### 2.1. Materials and Preparation

Acrylamide (AM), N,N-methylenenebisacrylamide (MBA), ammonium persulphate (APS), chitosan (CS), acetic acid, sodium chloride (NaCl), and deionized water were used to synthesis hydrogel. All the chemicals are analytically pure (AR) grade and from Sinopharm Chemical Reagent Co., Ltd. Portland cement from P.O. 52.5 (Huaxin Cement Co., Ltd. Wuhan, China) and standard sand were used to make cement paste.

**Figure 1:**
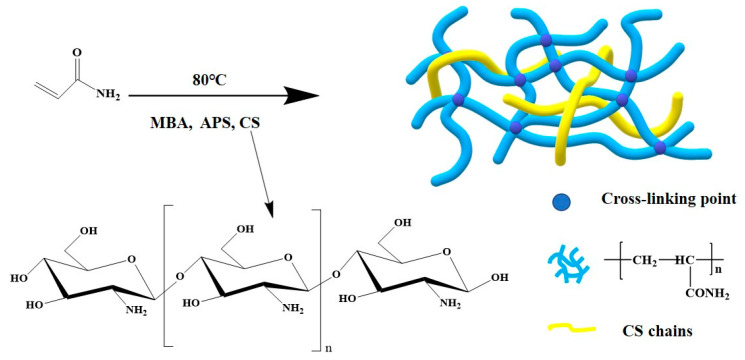
Preparation process of PAMC gel.

The preparation of hydrogels was as follows: 0.05 g chitosan was dissolved in 5 mL of 2% acetic acid solution and stirred for 4 h at 60 ℃ to obtain solution A; 2.5 g acrylamide (AM), quantitative N’N-methylenebisacrylamide (MBA), and 10 mg ammonium persulfate (APS) were dissolved in 5 mL of 2% acetic acid solution and stirred well to obtain solution B; then, solution B was quickly added into solution A and stirred 10 min to obtain the reaction solution, which was then put into a mold and sealed with cling film and placed in an oven at 80 ℃ for 3 h. Then, the polyacrylamide–chitosan hydrogel (PAMC) was obtained. Different ratios of PAMC are shown in Table 1, and the preparation process is shown in Figure 1.

### 2.2. Characterization of PAMC Gel

The PAMC gel was made into a thin film and soaked in deionized water for 48 h for further purification, with water changes every 12 h. The soaked gel film was freeze-dried for 48 h to obtain a PAMC dry gel film sample. The samples were treated with platinum spray and then tested by scanning electron microscopy (SEM, JSM-7500F, JEOL, Tokyo, Japan) with an accelerating voltage of 5 kV. The IR pattern of gel samples was obtained by Fourier transform infrared spectra (FTIR, Nicolet 6700, Thermo Nicolet Corporation, Plainville, MA, USA), and the scanning range was from 4000 cm^−1^ to 400 cm^−1^. Elemental analyses of the PAMC gel were conducted with an energy dispersive spectrometer (EDS, X-Max N80, Oxford Instruments, London, UK).

The PAMC gels were soaked in 2% acetic acid solution for 48 h and then dried under vacuum at 40 °C to a constant weight after ethanol extraction. The gel content (G_c_) was calculated according to Equation (1).

(1) $$G_{c}=\frac{m_{0}-m_{d}}{m_{0}}\times 100\%$$

where G_c_ is the swelling degree, m_0_ is the initial weight of the PAMC gel, and m_d_ is the weight of the dried PAMC gel.

The PAMC gels were immersed in sodium chloride solutions of different pH values/simulated seawater. The gels were removed at specific time intervals and weighed after drying the surface water with filter paper until the dissolution equilibrium. The swelling degree of the PAMC gel was calculated by Equation (2):

(2) $$S_{d}=\frac{m_{0}-m_{q}}{m_{0}}\times 100\%$$

where S_d_ is the swelling degree, m_0_ is the initial weight of the PAMC gel, and m_q_ is the weight of the PAMC gel at swelling equilibrium.

### 2.3. Isothermal Adsorption Model

Different concentrations of NaCl salt solutions were prepared: 0.002, 0.005, 0.01, 0.02, 0.04, 0.06, 0.08, 0.1, 0.2, and 0.3 mol/L. NaCl salt solutions (15 mL) of different concentrations were placed in sealed glass bottles, and 0.5 g of PAMC-1 was added, and after 30 h of oscillating adsorption, the concentration changes were measured by a PXSJ-216F ion meter to determine the concentration change. To better illustrate the adsorption mechanism of PAMC, the non-linear fitting of the Langmuir model [24,25] and the Freundlich model [26] were used to simulate the adsorption in this paper. The amount of chloride bound in PAMC was calculated using Equation (3):

(3) $$W=\frac{M\left(C_{0}-C\right)V}{m}$$

where W is the adsorption content of PAMC (mg/g), M is the relative mass fraction of Cl, V is the volume of solution (L), C_0_ is the initial concentration of solution (mol/L), C is the concentration after adsorption (mol/L), and m is the mass of PAMC gel.

### 2.4. Adsorption Behavior of PAMC in Solution

#### 2.4.1. Effects of AM and MBA Contents on the Adsorption Performance of PAMC

To study the chloride ion adsorption capacity of PAMC with different AM content or MBA content in solutions, simulated seawater was prepared according to ASTM D1141-1998 [27] for adsorption testing, and the chloride ion content was measured with a ion meter(PXSJ-216F, Shanghai Yidian Scientific Instruments Corporation, Shanghai, China).

First, 1.5 g PAMC gels (Table 1) were individually put into a sealed glass bottle with 15 mL of simulated seawater solution with a blank control group. Then, 10 mL of the adsorbed solution in the glass bottle was taken into a beaker with a pipette and diluted to 50 mL, and the chloride ion content of the diluted solution in the beaker was measured by an PXSJ-216F ion meter.

#### 2.4.2. Effect of pH on Adsorption Performance

Equal ionic concentrations of buffer solutions with pH = 4, 6.86, 9.18, or 12 were prepared, and an appropriate amount of NaCl was added to obtain 0.3 mol/L of NaCl solution with varying pH values. Then 1.5 g of PAMC gels with different BIS contents were respectively placed in 15 mL of the above NaCl solution for 48 h, and their concentration changes were tested by a PXSJ-216F ion meter compared to the control group.

### 2.5. Adsorption Behavior of PAMC in Simulated Sea Sand

In order to study the adsorption capacity of PAMC gels for chloride ions in a simulated sea sand environment, this paper formulated simulated sea sand according to a sand:seawater:PAMC ratio of 6:1:0.2 to conduct adsorption tests.

Firstly, 150 g of standard sand was weighed in a 250 mL beaker, 25 g of simulated seawater solution was added and stirred well, and 70 g of sea sand was taken out as a blank control group; the PAMC gels in Table 1 were cut into small particles of uniform size, and then 3 g of gel particles was added to 105 g of simulated sea sand, stirred well, and sealed for 72 h. Then the gels were picked out, 15 g of adsorbed sea sand was put into a beaker with 50 g of water and stirred for 24 h. 15 g of sea sand were taken from 70 g of the blank control group, placed into a beaker with 50 g of water and stirred for 24 h. The chloride ion content of the solutions in the above beakers was measured by a PXSJ-216F ion meter. Each of the above experiments was repeated three times.

### 2.6. Chloride Penetration Resistance in Cement Paste

In order to study the ability of gels to resist chloride ion in cement paste, this paper prepared net slurry sample blocks according to the water:cement ratio of 0.4 and the gel admixture of 1.06 wt%; the detailed contents are shown in Table 2. The well-mixed cement mixtures according to Table 2 were poured into 20 × 20 × 20 mm cement sample molds, and the molds were wrapped with cling film and cured at room temperature for 24 h. Then, the cured cement samples were demolded and sealed with paraffin wax, leaving only two opposite sides, and put into a saturated calcium hydroxide solution containing 5% mass fraction of sodium chloride at 25 °C. They were cured for 14 d and 28 d and then removed, dried, and placed in an oven for 2 h. The samples were then cut into 0–2.5 mm, 2.5 mm–5 mm, 5 mm–7.5 mm, and 7.5 mm–10 mm pieces at 2.5 mm intervals from the permeate surface, and then these pieces were ground into powder and sieved to remove the gel. The obtained powder was immersed in quantitative water and soaked for 24 h. The content of chloride ions in the soaking solution was tested with an ion meter, and then the content of chloride ions at different depths on the cement surface could be obtained.

## 3. Results and Discussion

### 3.1. Composition and Morphology of PAMC

**Figure 2:**
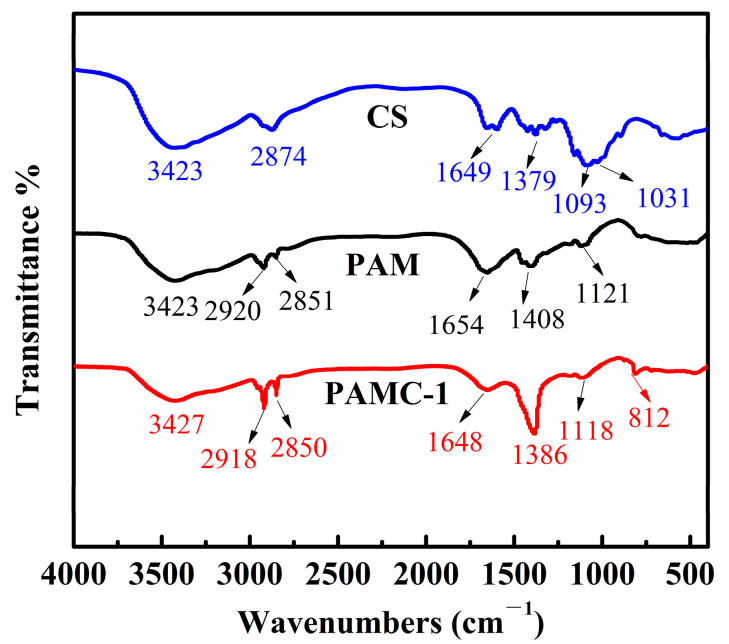
FTIR spectra of PAMC, PAM, and CS.

Figure 2 presented the FTIR patterns of CS, PAM, and PAMC. As shown in the spectra of CS, the broad peak at 3423 cm^−1^ was due to the stretching vibrations of -NH_2_ and -C-H groups [28,29,30], and the peaks at 2874 cm^−1^, 1093 cm^−1^, and 1031 cm^−1^ were related to the stretching vibrations of -C-H and C-O of C_6_ and -C-OH of C_3_ in chitosan, respectively [31]. The peak at 1649 cm^−1^ also corresponded with the Ⅰadsorption band of amide, while the peaks at 3427 cm^−1^, 2920cm^−1^ and 2851cm^−1^, and 1121cm^−1^ were related to the stretching vibrations of -NH_2_, -C-H, and -C-N in PAM chains, and the peak at 1654 cm^−1^ corresponded to the Ⅰ adsorption band of amide [32]. In the spectra of PAMC, the peaks at 3427 cm^−1^ were related to the stretching vibrations of -NH_2_ and -OH, the peak at 1648 cm^−1^ was due to the flexural vibration of C=O, and the peaks at 2918 cm^−1^ and 1118 cm^−1^ were related the stretching vibrations of -CH and C-O, respectively. The carbonyl absorption peak at 1648 cm^−1^ in PAMC was shifted to a lower frequency position compared to CS and PAM, which may be due to intermolecular or intramolecular hydrogen bonding interactions [28]. In addition, those peaks became more intensive due to the overlap of the groups in CS and PAM, which indicated coexistence of polyacrylamide and chitosan and the formation of a semi-interpenetrating network.

**Figure 3:**
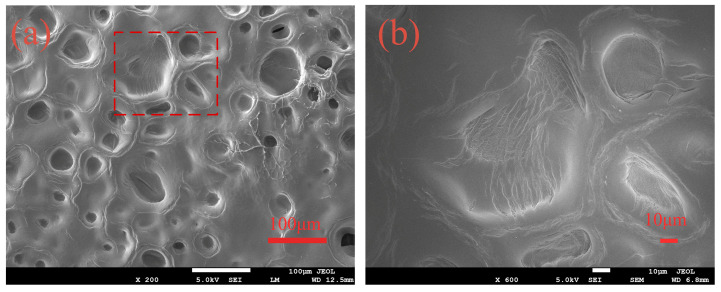
SEM image of hydrogels: (**a**) PAMC; (**b**) the partial enlarged detail of (**a**).

Figure 3 depicts the surface morphology of the PAMC gel before and after the adsorption of chloride ions. As seen in Figure 3a, there were many holes on the surface of the PAMC hydrogel; this was because the PAMC hydrogel was a porous three-dimensional network material with good swelling properties. Figure 3b shows an enlarged view of the rectangular area in Figure 3a. The fibrous structure of the gel can be clearly seen because the PAMC gel synthesized in this paper had a high monomer concentration, and more chitosan was added, so the gel network formed was relatively tight. This porous three-dimensional network structure enabled PAMC gels with good adsorption properties.

### 3.2. Adsorption in Solution

#### 3.2.1. Adsorption in Different Concentrations of NaCl Solution

The adsorption capacity of the PAMC gel in different concentrations of sodium chloride solution was studied. The adsorption isotherm equation was fitted according to the experimental data, and the adsorption mechanism of PAMC on chloride ions was clarified. As useful mathematical tools, adsorption isotherms can describe the adsorption type and intensity between adsorbent and adsorbate as well as show the adsorption level between the solid phase and the ion with different concentrations of solution.

The Langmuir isotherm in the liquid phase usually represents the solute adsorbed as a single molecular layer on the adsorption site on the surface of the adsorbent with the equation shown in Equation (4):

(4) $$W=\frac{W_{m}K_{L}C}{1+K_{L}C}$$

The linear relationship can be expressed mathematically by Equation (5) as follows:

(5) $$\frac{C}{W}=\frac{1}{K_{L}W_{m}}+\frac{1}{W_{m}}C$$

where K_L_ is the Langmuir constant; W_m_ is the maximum adsorption capacity of the adsorbent; and C is the equilibrium concentration of the adsorbate. 

The Freundlich model assumes that multilayer adsorption occurs on non-homogeneous surfaces and is an empirical model with the equation shown in Equation (6):

(6) $$W=K_{F}C^{\frac{1}{n}}$$

Taking the logarithm of both sides, linear Equation (7) is obtained:

(7) $$\mathrm{lgW}={\mathrm{lgK}}_{F}+\frac{1}{n}\mathrm{lgC}$$

where K_F_ and n are the Freundlich constant and the heterogeneity factor, respectively. 

**Figure 4:**
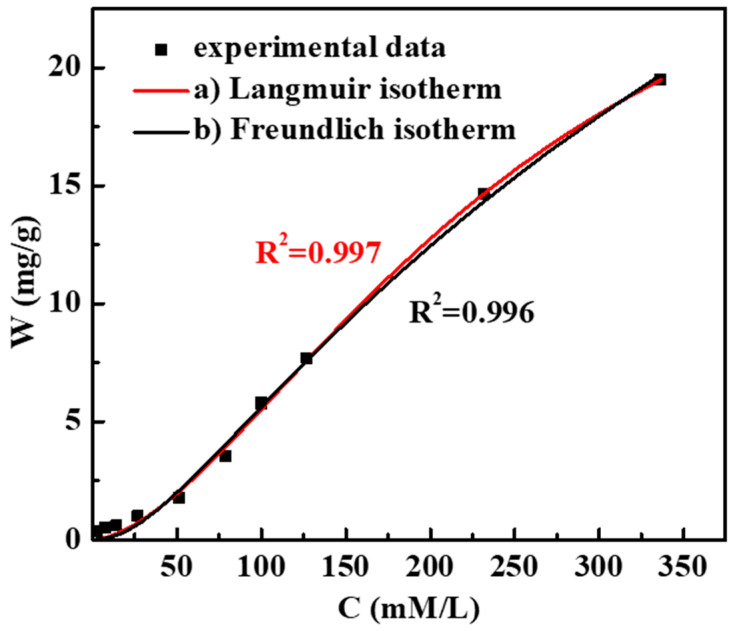
Isotherm of chloride adsorption by PAMC in NaCl solution at 25 ℃.

The adsorption isotherms of PAMC-1 at 25 °C are shown in Figure 4, from which it can be seen that both the Langmuir adsorption isotherm and the Freundlich adsorption isotherm were very close to the experimentally obtained data, with correlation indices of R_a_^2^ = 0.997 and R_b_^2^ = 0.996, indicating that the Langmuir model better matched the data of the real adsorption experiment. Therefore, the Langmuir model was chosen to represent the adsorption process of the PAMC gel; the surface layer of the PAMC gel possessed specific anion adsorption sites, which were uniformly distributed between the layers, and each adsorption site was occupied with equal probability [33]. The adsorption equilibrium was reached when the number of chlorides entering the adsorption sites was equal to the number of chlorides leaving the adsorption sites. 

#### 3.2.2. Adsorption in Simulated Seawater

**Figure 5:**
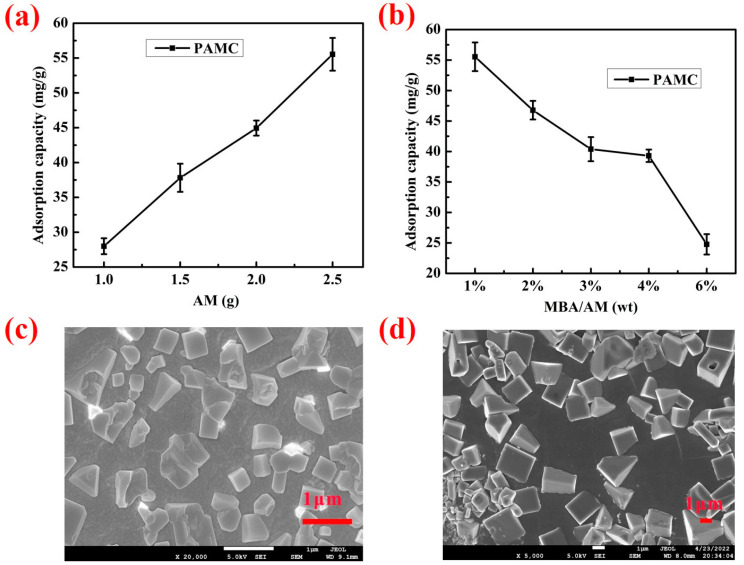
Effect factors of adsorption capacity of PAMC: (**a**) AM content; (**b**) MBA content; (**c**) surface morphology; and (**d**) internal morphology of PAMC-1 absorbed in seawater.

In order to extract the gel network of PAMC, we soaked it with acetic acid and then ethanol to obtain the gel content, as shown in Table 1. The gel content (Gc) of PAMC with different ratios was 85%–95% of the weight of the initial mixture, which means the formation of a gel network. Figure 5 shows the effect of monomer content and cross-linker content on the adsorption of PAMC gels in a seawater environment. PAMC was found to be a network structure composed of polyacrylamide chains and chitosan chains together, and it possessed -OH and -CONH- groups. O and N in -OH and -CONH- could form hydrogen bonds with chlorine, and studies have shown that -NH could form strong hydrogen bonding interactions with chlorine [34], so it could effectively adsorb chloride ions. From Figure 5a it can be seen that the adsorption capacity of PAMC for chloride ions increased continuously with increases in the monomeric acrylamide content. The adsorption capacity of PAMC-1 was increased by 98.3% compared with that of PAM_1_C. This was due to the fact that with the increase of acrylamide content, the amide groups in the gel network also increased, increasing the adsorption sites of PAMC for chloride ions. As shown in Figure 5b, the adsorption capacity of PAMC for chloride was gradually decreased with increases of the crosslinker content. This was due to the fact that the gel was a three-dimensional network structure with many pores between it, and the increase of the crosslinker content enhanced the crosslinking density and crosslinking strength of the polymer chains, thereby reducing the swelling and pores between the networks, making it difficult for the chloride ion to enter. 

When the crosslinker content was 1% of the AM content, the adsorption capacity was a maximum of 55.53 mg/g, and the decrease rate of PAMC adsorption became faster at 4%–6%, likely because the crosslinking reaction occurred between excessive crosslinker and chitosan, which would not only reduce the flexibility of the chitosan chains in the gel network but also reduce the amino adsorption sites on the chitosan chains. 

The scanning electron microscope (SEM) image of the freeze-dried PAMC gel immersed in simulated seawater is shown in Figure 5c,d. It can be seen that stereoscopic structures of different sizes were uniformly distributed on the surface of the gel. These stereoscopic structures were the crystallization of salts in simulated seawater, the main component of which was sodium chloride, indicating that the gel adsorbs chloride ions in simulated seawater.

**Figure 6:**
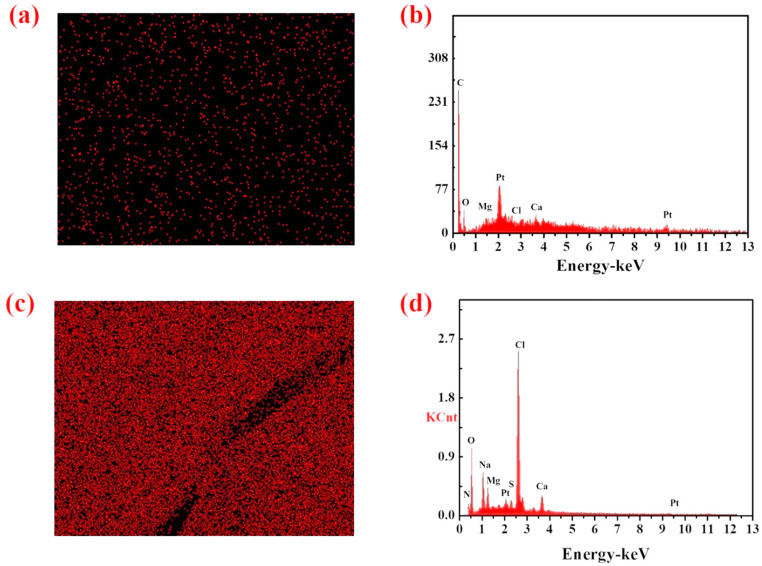
The chloride content and elemental distribution in PAMC gels: (**a**,**b**) before adsorption; (**c**,**d**) after adsorption in seawater.

Figure 6a,c show the surface distribution of chlorine measured in the PAMC before and after adsorption in the simulated seawater, and the red dots indicate the presence of chlorine. It can be seen that the red dots in Figure 6a are few and sparse, which might be caused by the noise in the working area [35], indicating that the PAMC gel before adsorption contained almost no chlorine element. Compared with Figure 6a, the distribution of red dots in Figure 6c is uniform and dense, indicating that there were more chloride ions in the adsorbed PAMC gel. This indicated that the PAMC gel effectively adsorbed chloride ions in simulated seawater due to the swelling effect and hydrogen bonding of amide groups with chloride ions. The relative element intensity in PAMC corresponding to Figure 6a,c is shown in Figure 6b,d, respectively. As shown in Figure 6b, the relative element intensity of Cl was hardly seen, and the relative element intensity of carbon was high, which was the carbon in the long chain of polyacrylamide, while the relative element intensity of chlorine shown in Figure 6d was high, which explained the fact that the PAMC gels could effectively adsorb the chloride ions in solution. 

#### 3.2.3. Effect of pH on the Adsorption of PAMC

**Figure 7:**
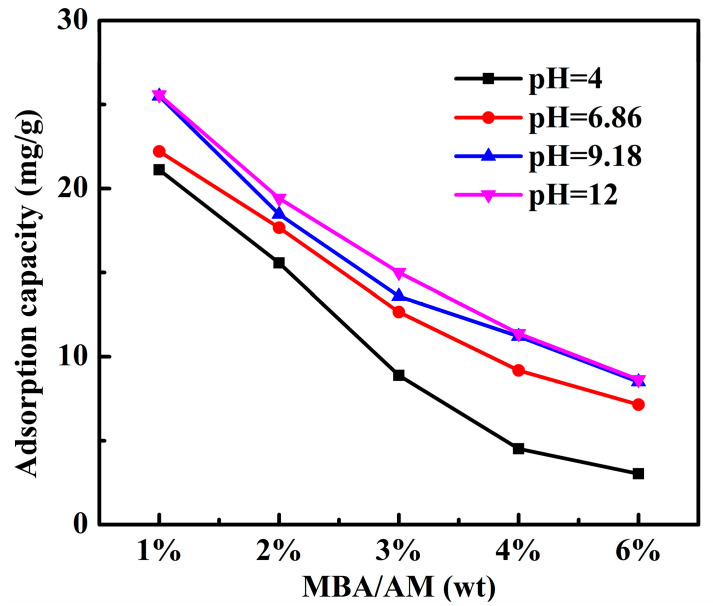
Effect of pH on adsorption capacity of PAMC.

The effect of pH on adsorption capacity of PAMC is displayed in Figure 7. The adsorption of PAMC showed a decreasing trend with increases in the crosslinker content, and higher adsorption performance was obtained for PAMC-1 under different pH conditions, which was consistent with the previous description. Furthermore, the effect of pH on the adsorption performance of PAMC first increased and then decreased with increases in the crosslinker content. PAMC gels were synthesized using acetic acid as the solvent, as the acetic acid molecules wrapped in the gel network would prevent the gel network from stretching and swelling under acidic conditions. Since PAMC-1 had the lower crosslinkage and the most flexible network, although it was hindered by acetic acid, the pores between its network could partially offset this effect. With increases in the crosslinker content, the flexibility of the gel network decreased, the cross-link density increased, and the void became smaller, which was not enough to eliminate the effect of insufficient swelling, so the effect of pH on PAMC-2, PAMC-3, and PAMC-4 gradually increased. As the crosslinker content continued to increase to excessiveness, the crosslink density of PAMC-6 was overdosed, and the degree of swelling was low, so that the acetic acid wrapped in the gel network had little effect on it. 

The equilibrium swelling degree of PAMC-1 in NaCl solutions with different pH values is shown in Table 3. It can be seen from Table 3 that the swelling degree of the PAMC gel at pH = 12 was slightly higher than that at pH = 4. However, the adsorption capacity of chloride ions on the PAMC gel at pH = 4 was lower than that at other pH conditions. This might be because the amino group in the PAMC structure would be protonated in a strong acidic environment, so that it could electrostatically interact with anions in solution. Furthermore, this allowed phosphate ions in the constituent pH buffer solution to compete with chloride ions for adsorption sites. Therefore, the adsorption amount of chloride ions on the PAMC gel at pH = 4 decreased slightly. The maximum adsorption content of PAMC-1 was 26.27 mg/g at pH = 12, indicating that the PAMC gels could be effectively used in alkaline environments.

### 3.3. Adsorption in Sea Sand

**Figure 8:**
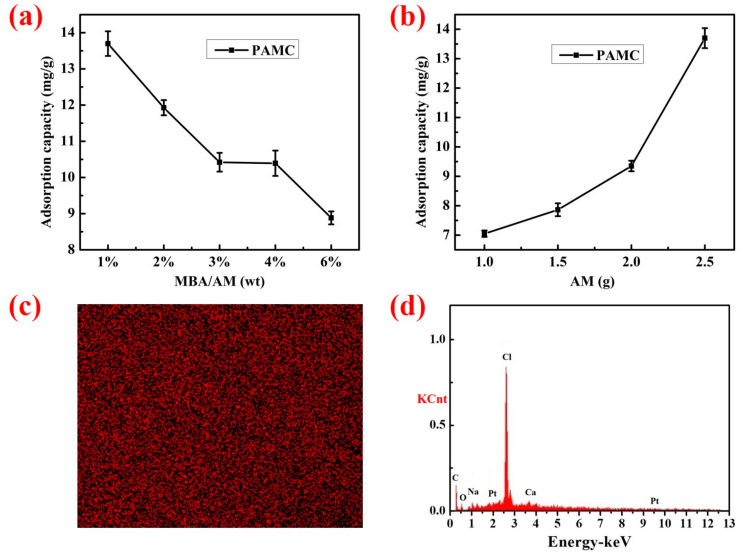
Effect of MBA (**a**) and AM (**b**) content on adsorption capacity of PAMC in the sea sand environment; the chloride content (**c**) and elemental distribution (**d**) in PAMC gels after being adsorbed in sea sand.

The effects of monomer content and cross-linker content on the adsorption of PAMC gels in a sea sand environment are shown in Figure 8a,b, respectively. The concentration of chloride ions in the sea sand before and after adsorption was measured by an ion meter, and the average mass of chloride ions that could be adsorbed per gram of gel was calculated. As shown in Figure 8a, the ability of the gel to adsorb chloride ions in the simulated sea sand environment gradually increased with the increase of acrylamide content, which was because the increase of acrylamide content provided more chloride ion adsorption sites, which was consistent with the description in Figure 5. As shown in Figure 8b, the ability of the gel to adsorb chloride ions in the simulated sea sand gradually decreased with the increase of the crosslinker content, which was due to the crosslinking density being enhanced with the increase of the crosslinker content, which made the PAMC gel network more compact, less flexible, and more difficult to swell. Therefore, the channels for chloride ions to enter the interior of the gel became narrower, and the adsorption amount decreased. 

The 1% MBA content of the PAMC gels obtained the maximum adsorption capacity of 13.7 mg/g in the simulated sea sand environment. Compared with what was shown in Figure 5, the adsorption amount of PAMC gels in simulated sea sand was lower and less influenced by the content of the crosslinker. This was probably because the concentration of chloride ions in the simulated sea sand was lower than that in the simulated seawater, as well as the very small amount of solution that existed in the simulated sea sand, leading to the reduction of chloride ions captured by gel swelling.

The surface distribution of Cl measured in the PAMC after adsorption in the simulated sea sand and the relative element intensity are shown in Figure 8c,d, respectively. It can be seen from Figure 8c that the red dots were uniformly and densely distributed, indicating that the gel contained a lot of chloride ions, which proved that the PAMC could effectively capture the chloride ions in the simulated sea sand. In the corresponding Figure 8d, the relative intensity of Cl was higher, which again proved that the PAMC gel could effectively adsorb the chloride ions in the simulated sea sand. In addition, compared with the blank group, the concentration of chloride ions in the sea sand after adsorption by PAMC-1 decreased by 65.41%.

### 3.4. Chloride Penetration Resistance in Cement Paste

**Figure 9:**
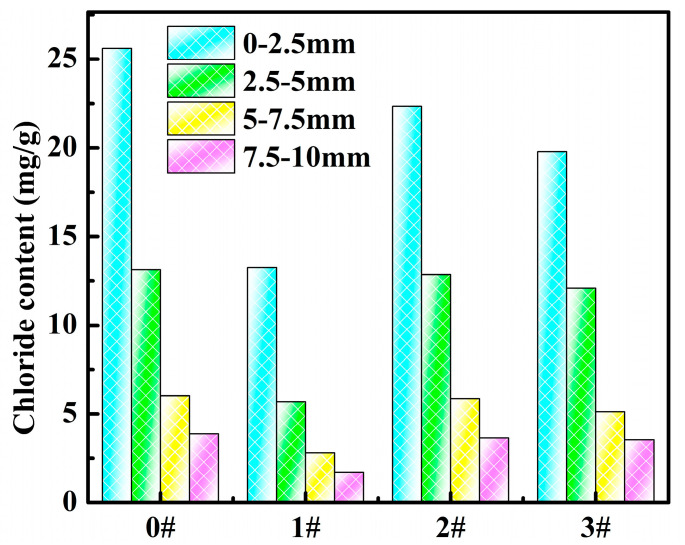
Mass fraction of chloride ions at different depths in mortar samples cured for 14 d (0^#^: net slurry, 1^#^: 1.06wt% PAMC-1+net slurry, 2^#^: 1.06wt% PAMC-3+net slurry, 3^#^: 1.06wt% PAMC-6+net slurry).

**Figure 10:**
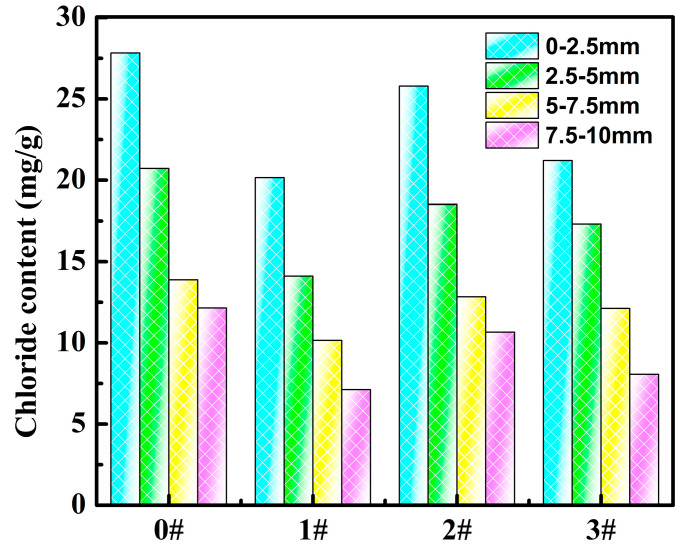
Mass fraction of chloride ions at different depths in mortar samples cured for 28 d (0^#^: net slurry, 1^#^: 1.06wt% PAMC-1+net slurry, 2^#^: 1.06wt% PAMC-3+net slurry, 3^#^: 1.06wt% PAMC-6+net slurry).

The free chloride ion mass fractions at different depths from the surface of the cement paste after curing for 14 d and 28 d are displayed in Figure 9 and Figure 10, respectively. It can be seen from Figure 9 that the chloride ion content varied at different depths from the permeation surface because it took some time for the chloride ions to enter the cement interior by permeation, and the cement matrix itself could play a role in hindering chloride ion permeation. The chloride ion content at each depth of blank group 0^#^ was higher than that of the other groups, indicating that the PAMC gels could effectively stop the chloride ion penetration process in cement and improve the chloride ion penetration resistance of cement. This was consistent with the adsorption results of the PAMC gels in simulated sea sand and seawater because the amide groups and hydroxyl groups in the PAMC gel could form hydrogen bonds with chloride ions to provide adsorption sites, and the swelling effect of the gel could also capture chloride ions. The 1^#^ had the lowest chloride ion content at each depth, compared with 0^#^, 0–2.5 mm was reduced by 48.2%, and 7.5–10 mm was reduced by 55.8%, which indicated that the PAMC-1 gel added in the net slurry could effectively improve the chloride ion penetration resistance of cement. The overall improvement effect of 2^#^ and 3^#^ were not obvious; the chloride ion content of the depth at 0–2.5 mm was reduced by 12.7% and 22.7%, respectively. In addition, the chloride ion contents at all depths of 3^#^ were lower than those of 2^#^, indicating that the addition of PAMC-6 on the improvement of cement resistance to chloride ion permeability was better than the addition of PAMC-3. 

As shown in Figure 10, the chloride ion contents at all depths in the samples cured for 28 d increased significantly compared to those of samples cured for 14 d. This was due to the limited ability of concrete to cure chloride ions, which slowly penetrated into the cement paste with time. The chloride ion contents at all depths of the 1^#^ sample were lowest, and the chloride ion contents at 0–2.5 mm and 7.5–10 mm compared with the blank group were reduced by 27.6% and 41.4%, respectively. The chloride ion contents at 7.5–10 mm of 2^#^ and 3^#^ samples were reduced by 12.3% and 33.6%, respectively. As a result, the anti-chloride ion permeation ability of cement paste was ranked as follows: 1^#^>3^#^>2^#^>0^#^, which indicated that the PAMC-1 gel could most effectively impede the permeation of chloride ions, and the PAMC-6 gel could also impede the permeation of chloride ions, while the PAMC-3 gel could only play a small part in the anti-chloride ion permeation process. Those results were consistent with what is depicted in Figure 8. Compared with Figure 8, the chloride ion contents at 0–2.5 mm were closer between 3# and 1# in Figure 10, which was probably because the chloride ion content at 0–2.5 mm was higher with the increase of the curing time, and the adsorption capacity of the PAMC gels for chloride ions here reached saturation. The chloride ions entering and exiting the PAMC-1 gel network reached dynamic equilibrium. Although the adsorption capacity of the PAMC-1 gel was higher than that of PAMC-6, the PAMC-6 gel network was denser, and the chloride ions could not escape easily. Thus, the curing capacity of the PAMC-6 gel for chloride ions in this environment was close to that of the PAMC-1 gel.

## 4. Conclusions

Based on the swelling properties conferred by the gel network structure and the hydrogen bonding between the polymer functional groups and the solute, a polyacrylamide–chitosan semi-interpenetrating network hydrogel was synthesized in this paper, and the properties of the PAMC gel were characterized by FTIR and SEM. The data from the adsorption of chloride ions in solution by PAMC gels well fitted the Langmuir isothermal model and had physical adsorption with a theoretical adsorption capacity of up to 58 mg/g. With an increase of AM content or a decrease of MBA content, the adsorption capacity of chloride ions of PAMC was gradually increased. The PAMC gel had a higher adsorption capacity in alkaline environments and was suitable for strongly alkaline cement-based materials. The best chloride ion permeation resistance of cement occurred when 1.06 wt% of PAMC-1 gel was incorporated into the cement, which was 55.8% higher compared to the blank group.

## Figures and Tables

**Table 1:** Specific ratios and properties of PAMC hydrogel.

Samples	AM	MBA/AM	CS	APS	G_c_	S_d_
	(g)	(wt%)	(g)	(g)	(wt%)	(wt%)
PAM_1_C	1	1	0.5	0.01	91 ± 3.6	188.1
PAM_1.5_C	1.5	1	0.5	0.01	87 ± 2.3	186.0
PAM_2_C	2	1	0.5	0.01	87 ± 1.5	178.4
PAMC-1	2.5	1	0.5	0.01	84 ± 2.6	176.5
PAMC-2	2.5	2	0.5	0.01	90 ± 1.5	121.8
PAMC-3	2.5	3	0.5	0.01	88 ± 1	92.7
PAMC-4	2.5	4	0.5	0.01	90 ± 1.5	70.9
PAMC-6	2.5	6	0.5	0.01	93 ± 0.6	46.5

**Table 2:** Specific ratios of concrete samples.

Sample	Content (g)				
	Water	P.O.52.5	PAMC-1	PAMC-3	PAMC-6
0	80	200	-	-	-
1	80	200	3	-	-
2	80	200	-	3	-
3	80	200	-	-	3

**Table 3:** Equilibrium swelling degree of PAMC-1 in NaCl solutions with different pH values.

PAMC-1	pH = 4	pH = 6.86	pH = 9.18	pH = 12
Swelling degree (%)	174	165	170	178

## References

[1] Zhang L., Khan N., Pu C.S. (2019). A new method of plugging the fracture to enhance oil production for fractured oil reservoir using gel particles and the HPAM/Cr3+ system. Polymers.

[2] Zhou Y.M., Li T., Shen J.L., Meng Y., Tong S.H., Guan Q.F., Xia X.X. (2021). Core-shell structured magnetic carboxymethyl cellulose-based hydrogel nanosorbents for effective adsorption of methylene blue from aqueous solution. Polymers.

[3] Quezada G.R., Rozas R.E., Toledo P.G. (2021). Polyacrylamide adsorption on (101) quartz surfaces in saltwater for a range of pH values by molecular dynamics simulations. Miner. Eng..

[4] Zou W.J., Gong L., Huang J., Pan M.F., Lu Z.Z., Sun C.B., Zeng H.B. (2019). Probing the adsorption and interaction mechanisms of hydrophobically modified polyacrylamide P(AM-NaAA-C_16_DMAAC) on model coal surface: Impact of salinity. Miner. Eng..

[5] Binaeian E., Zadvarzi S.B., Yuan D. (2020). Anionic dye uptake via composite using chitosan-polyacrylamide hydrogel as matrix containing TiO_2_ nanoparticles; comprehensive adsorption studies. Int. J. Biol. Macromol..

[6] Hong T.T., Okabe H., Hidaka Y., Omondi B.A., Hara K. (2019). Radiation induced modified CMC-based hydrogel with enhanced reusability for heavy metal ions adsorption. Polymer.

[7] Moon S.J., Jang S.W., Kim Y.R., Gil M., Lee K.J. (2020). Prussian blue decorated hydrogel particles for effective removal of cesium ion from aqueous media. Polymer.

[8] Zou X.Q., Zhang H., Chen T., Li H.T., Meng C.H., Xia Y., Guo J. (2019). Preparation and characterization of polyacrylamide-e/sodium alginate microspheres and its adsorption of MB dye. Colloids Surf..

[9] Feng L.H., Zhang Q., Ji F.Y., Jiang L., Liu C.C., Shen Q.S., Liu Q. (2020). Phosphate removal performances of layered double hydroxides (LDH) embedded polyvinyl alcohol/lanthanum alginate hydrogels. Chem. Eng. J..

[10] Xi H., Li Q.Q., Yang Y., Zhang J.F., Guo F., Wang X.G., Xu S.K., Ruan S.P. (2021). Highly effective removal of phosphate from complex water environment with porous Zr-bentonite alginate hydrogel beads: Facile synthesis and adsorption behavior study. Appl. Clay Sci..

[11] Yu X.L., Zhang J., Zheng Y. (2021). Perchlorate adsorption onto epichlorohydrin crosslinked chitosan hydrogel beads. Sci. Total Environ..

[12] Qing Z.L., Wang L.J., Liu X.Y., Song Z.W., Qian F., Song Y.H. (2022). Simply synthesized sodium alginate/zirconium hydrogel as adsorbent for phosphate adsorption from aqueous solution: Performance and mechanisms. Chemosphere.

[13] Han S.W., Zhong J., Ding W.J., Ou J.P. (2021). Strength, hydration, and microstructure of seawater sea-sand concrete using high-ferrite Portland cement. Constr. Build. Mater..

[14] Costa A., Júlio A. (2002). Case studies of concrete deterioration in a marine environment in Portugal. Cem. Concr. Compos..

[15] Wang X.X., Liu J.P., Jin Z.Q., Chen F.X., Zhong P.H., Zhang L. (2022). Real-time strain monitoring of reinforced concrete under the attacks of sulphate and chloride ions based on XCT and DIC methods. Cem. Concr. Compos..

[16] Kenny A., Katz A. (2020). Steel-concrete interface influence on chloride threshold for corrosion—Empirical reinforcement to theory. Constr. Build. Mater..

[17] Xiao L.F., Chen D.Q., Jiang M.J., Xiao L., Mei G.X. (2021). Experimental and numerical analysis of chloride transport in finite concrete under reverse water pressure. Constr. Build. Mater..

[18] Li W.J., Guo L. (2021). Peridynamic investigation of chloride diffusion in concrete under typical environmental factors. Ocean Eng..

[19] Yang C.Y., Li L., Li J.P. (2020). Service life of reinforced concrete seawalls suffering from chloride attack: Theoretical modelling and analysis. Constr. Build. Mater..

[20] Li G., Ding Y., Gao T.Y., Qin T.M., Lv Y.J., Wang K.J. (2021). Chloride resistance of concrete containing nanoparticle-modified polymer cementitious coatings. Constr. Build. Mater..

[21] Bhogone M.V., Subramaniam K.V.L. (2022). Enhanced concrete performance in cracking resistance and chloride penetration using hybrid fiber blends. Mater. Today Proc..

[22] Carmona J., Garcés P., Climent M.A. (2015). Efficiency of a conductive cement-based anodic system for the application of cathodic protection, cathodic prevention and electrochemical chloride extraction to control corrosion in reinforced concrete structures. Corros. Sci..

[23] Luján V.A.F., Rangel J.M.M., Quero V.G.J., García P.M. (2021). Chloride-binding capacity of ternary concretes containing fly ash and untreated sugarcane bagasse ash. Cem. Concr. Compos..

[24] Hu Q.L., Liu Y., Feng C.P., Zhang Z.Y., Lei Z.F., Shimizu K. (2018). Predicting equilibrium time by adsorption kinetic equations and modifying Langmuir isotherm by fractal-like approach. J. Mol. Liq..

[25] Rosly N.Z., Ishak S., Abdullah A.H., Kamarudin M.A., Ashari S.E., Ahmad S.A.A. (2021). Fabrication and Optimization Calix[8]arene-PbS Nanoadsorbents for the Adsorption of Methylene Blue: Isotherms, Kinetics and Thermodynamics Studies. J. Saudi Chem. Soc..

[26] Araújo C.S.T., Almeida I.L.S., Rezende H.C., Marcionilio S.M.L.O., Léon J.J.L., Matos T.N. (2018). Elucidation of mechanism involved in adsorption of Pb(II) onto lobeira fruit (*Solanum lycocarpum*) using Langmuir, Freundlich and Temkin isotherms. Microchem. J..

[27] (2003). Standard Practice for the Preparation of Substitute Ocean Water.

[28] Saha S., Sarkar P. (2012). Arsenic remediation from drinking water by synthesized nano-alumina dispersed in chitosan-grafted polyacrylamide. J. Hazard. Mater..

[29] Li X., Zheng H.L., Wang Y.L., Sun Y.J., Xu B.C., Zhao C.L. (2017). Fabricating an enhanced sterilization chitosan-based flocculants: Synthesis, characterization, evaluation of sterilization and flocculation. Chem. Eng. J..

[30] Li K., Wang Y.W., Huang M., Yan H., Yang H., Xiao S.J., Li A.M. (2015). Preparation of chitosan-graft-polyacrylamide magnetic composite microspheres for enhanced selective removal of mercury ions from water. J. Colloid Interface Sci..

[31] Viswanathan N., Sundaram C.S., Meenakshi S. (2009). Removal of fluoride from aqueous solution using protonated chitosan beads. J. Hazard. Mater..

[32] Liu Q., Xu K., Hu G.X., Zeng F.M., Li X., Li C., Zhang Y. (2022). Underwater super elastic MOF/polyacrylamide/chitosan composite aerogel for efficient 2, 4-dichlorophenoxyacetic acid adsorption. Colloids Surf. A.

[33] Zhang L., Sellaoui L., Franco D., Dotto G.L., Bajahzar A., Belmabrouk H., Petriciolet A.B., Oliveira M.L.S., Li Z.C. (2020). Adsorption of dyes brilliant blue, sunset yellow and tartrazine from aqueous solution on chitosan: Analytical interpretation via multilayer statistical physics model. Chem. Eng. J..

[34] Biswas S., Mallik B.S. (2020). Solvent-mediated dynamics and stretching profile of amide modes: QM/MM simulations of N-methylacetamide in ionic and various molecular liquids. J. Mol. Liq..

[35] Stoney D.A., Stoney P.L. (2022). Identification of factors affecting SEM/EDS analysis for discrimination and classification among common items of evidence using particle combination profiles. Forensic Sci. Int..

